# The Association between Genomic DNA Methylation and Diabetic Peripheral Neuropathy in Patients with Type 2 Diabetes Mellitus

**DOI:** 10.1155/2019/2494057

**Published:** 2019-11-03

**Authors:** Hong-Hong Zhang, Xingfa Han, Mengmeng Wang, Qingfang Hu, Sicheng Li, Meng Wang, Ji Hu

**Affiliations:** ^1^Department of Endocrinology, The Second Affiliated Hospital, Soochow University, Suzhou 215004, China; ^2^Department of Endocrinology, Suzhou Science and Technology Town Hospital, Nanjing Medical University, Suzhou 215004, China; ^3^Clinical Pharmacology Laboratory, The Second Affiliated Hospital, Soochow University, Suzhou 215004, China

## Abstract

**Aim:**

DNA methylation is thought to be involved in regulating the expression of key genes and inducing diabetic peripheral neuropathy (DPN). However, clinically, the level of whole-genome DNA methylation and its relationship with DPN remains unclear.

**Methods:**

186 patients with type 2 diabetes mellitus (T2DM) admitted to the Second Affiliated Hospital of Soochow University since Jul. 2016 to Oct. 2017 were enrolled in the study, including 100 patients in the DPN group and 86 patients in the non-DPN group, diagnosed with Toronto Clinical Scoring System (TCSS). Clinical and biochemical characteristics between the two groups were compared, and the correlations with TCSS scores were analyzed. Furthermore, the levels of genomic DNA methylation of leukocytes, measured with high-performance liquid chromatography-tandem mass spectrometry (LC-MS/MS), were also analyzed between the two groups.

**Results:**

Age, duration, triglyceride (TG), total cholesterol (TC), low-density lipoprotein (LDL-C), creatinine, uric acid (UA), blood urea nitrogen (BUN), and C-reactive protein (CRP) were significantly higher in the DPN group. Estimated glomerular filtration rate (eGFR) and the level of genomic DNA methylation were much lower in the DPN group. Spearman correlation analysis showed that TCSS was positively correlated with age, duration, UA, and CRP and was negatively correlated with body mass index (BMI), eGFR, and the level of genomic DNA methylation. Interestingly, multiple stepwise regression analysis showed that only duration, genomic DNA methylation, and eGFR had impacts on TCSS. The results also showed that the levels of genomic DNA methylation did not change significantly whether or not there was renal injury. Another multiple stepwise regression analysis showed that TCSS and BMI were the influencing factors of genomic DNA methylation. Finally, we found that genomic DNA methylation levels were decreased significantly in the DPN group compared with the non-DPN group when the duration is ≥5 years or BMI ≥ 25 kg/m^2^.

**Conclusion:**

Low level of genomic DNA methylation is a relative specific risk factor of diabetic peripheral neuropathy in patients with type 2 diabetes.

## 1. Introduction

According to the report of the World Health Organization (WHO), there are about 422 million people with diabetes in the world (https://www.who.int/news-room/fact-sheets/detail/diabetes), and the prevalence in adult is about 9.5% [1]. In China, the latest prevalence of diabetes in adult is about 10.4% [2]. The complications of diabetes are the main cause of death and disabilities, which bring people huge spiritual and economic burden. Diabetic peripheral neuropathy (DPN) is one of the most common complications. The clinical manifestations of DPN are mainly distal limb paresthesia and movement disorders. Paresthesia is usually characterized by peripheral limbs' numbness, burning and tingling pain, cold sensation, and formication, while movement disorders are characterized by moving weakly, inflexibly, and unsteadily. Severe DPN causes foot ulcer and amputation, seriously affecting life quality of the patients. Therefore, it is important to study the risk factors of DPN and to find out the potential therapeutic targets.

The electroneuromyography examination is the “gold standard” for diagnosing DPN. However, it only detects the function of large nerve fibers other than the small fibers with lower cost performance. Toronto Clinical Scoring System (TCSS) is an integrated scoring system based on the characteristics of DPN, and it can evaluate the function of myelinated and small unmyelinated nerve fibers. The sensitivity and specificity of TCSS are above 70% when the scores ≥6, which is suitable for clinical diagnosis of DPN and large-scale epidemiological investigation [3, 4].

DNA methylation is one of the epigenetic modifications, which refers to a methyl transfers from S-adenosylmethionine (SAM) to cytosine on CpG dinucleotide under the action of DNA methyltransferase (DNMTs). In the mammalian genome, this modification takes place on the fifth carbon atom of the cytosine base, forming 5-methylcytosine [5]. DNA methylation is related to the abnormal expression of genes, repairing of DNA damage, instability of genomes, and the change of genetic traits. It can change the chromatin structure and plays an important role in gene expression [6, 7]. Growing research showed that DNA methylation is related to the occurrence and development of many diseases, including T2DM and its complications [8, 9].

The present study mainly analyzed the risk factors of diabetic peripheral neuropathy, including genomic DNA methylation level of leucocytes, and analyzed their correlation with TCSS scores.

## 2. Research Design and Methods

### 2.1. Research Design

According to the criteria for admission, the clinical data, laboratory tests, and blood samples of patients with DPN were collected and analyzed, and diabetic patients without DPN were used as controls in this cross-sectional study.

### 2.2. Subjects

A total of 186 patients with type 2 diabetes older than 18 y were recruited from the endocrine department at the Second Affiliated Hospital of Soochow University from Jul. 2016 to Oct. 2017. The diagnosis of T2DM was based on the diagnostic criteria of WHO in 1999. Exclusion criteria are as follows: neuropathy caused by other reasons, such as intervertebral disc herniation, spinal canal stenosis, sciatica, cerebral infarction, giant cell anemia, Guillain-Barre syndrome, and lower limb occlusive vascular disease, trauma or surgery of lower limb, all kinds of severe acute and chronic inflammation, malignant tumor, chronic alcoholism, and other serious diseases. Moreover, patients with hyperthyroidism, hypothyroidism, and diabetic acute complications, such as hyperosmotic coma, diabetic ketoacidosis, and lactic acidosis, were excluded. The protocol of the present study was approved by the Institutional Review Board of the Second Affiliated Hospital of Soochow University, and the approving registration number is (2016) Ethics Review No. K11. Every patient was fully informed about the study and signed a written consent. The patients' personal privacy was kept strictly confidential: the sample data was stored electronically in a special computer with a password, which was only available to researchers. The refrigerator in which the samples were stored was a dedicated biological sample storage refrigerator, and the key was kept by the main researcher, and the blood samples could only be used for this protocol. The patients' medical records were kept in the hospital.

### 2.3. Clinical and Biochemical Measurements

Detailed information, such as age, gender, and medical histories, were recorded by trained physicians, and each patient received anthropometric measurements, including body weight and height measured in light clothes and bare feet. Body mass index (BMI) was calculated according to the formula: BMI (kg/m^2^) = weight (kg)/height (m)^2^.

TCSS was used to evaluate DPN, as previously described in details [10]. A senior technical staff who specialized in neurological examinations for more than 10 years was responsible for inquiring and testing according to the content of TCSS. Each patient was questioned about the presence or absence of pain (such as stabbing, burning, or shock-like pain), numbness, tingling, and weakness in the feet; the presence or absence of similar upper-limb symptoms; and the presence or absence of unsteadiness on ambulation. Sensory testing was performed at the first toe and rated as normal or abnormal. Patients were asked about their sensation while their toes were stimulated by needle, light touching, instrument with different temperature, tuning fork, and about their joint position sensation. Nerve reflexes of lower limbs including knee reflex and ankle reflex were tested, respectively. The outcome, the clinical neuropathy score, is a continuous variable ranging from a minimum of 0 (no neuropathy) to a maximum of 19 points. Six points are derived from symptoms, 5 from sensory testing distally at the toes, and 8 from lower limb reflexes. Patients with TCSS scores ≥6 were divided into the DPN group, and correspondingly, the scores <6 are considered to be no DPN.

All patients fasted for at least 8 hours before blood samples were collected. Biochemical parameters, such as FPG, TC, TG, HDL-C, LDL-C, UA, creatinine, BUN, and CRP, were measured with an automated biochemical instrument (Cobas8000-c702, Roche, Basel, Switzerland). eGFR was calculated by using the modified simplified MDRD equation. UACR was also tested to assess renal injury. HbA1c was detected with high-performance liquid chromatography (HPLC, BioRad Laboratories, Hercules, CA, USA). The level of fasting C-peptide was measured using electrochemiluminescence method (Roche Diagnostics GmbH, Mannheim, Germany).

### 2.4. Genomic DNA Methylation Detection

DNA was extracted from white blood cells using the HiPure Blood DNA Kits (D3111, Magen). The level of genomic DNA methylation was determined by LC-MS/MS (Agilent 1260-API 4000, USA), as previously described in details [11]. Cracking DNA with 200 *μ*l of 99% formic acid at 140°C for 90 min and then suspending fragment with 200 *μ*l water for LC-MS/MS analysis were done. The specific conditions of LC-MS/MS are shown in Supplementary [Supplementary-material supplementary-material-1]. The analyte was separated on a XTerra® RP18 column (4.6 × 150 mm, 3.5 *μ*m, Waters, Ireland). Column and autosampler temperatures were 30°C and 4°C, respectively. Mobile phases were composed of A (water) and B (methanol) using a gradient elution of 97%-97% (*v*/*v*) A at 0-5 min, 97%-0% A at 5-6 min, 0%-0% A at 6-7.5 min, 0%-97% A at 7.5-8 min, and 97%-97% A at 8-15 min with a flow rate set at 0.5 ml/min. The injection volume was 10 *μ*l. Mass spectrometric detection was performed on an API 4000 instrument (SCIEX, Ontario, Canada) equipped with an electrospray ionization (ESI) interface in the positive ion mode. The tandem mass spectrometer was operated under the multiple reaction monitoring modes (MRM) at m/z 112.1 → 95.1 and m/z 126.1 → 109.1 for Cyt and 5-mCyt, respectively. The declustering potential (DP) and collision energy (CE) of both analytes are 114 V and 10 V. The typical chromatographic peaks were obtained as shown in Supplementary [Supplementary-material supplementary-material-1]. Cytosine (Cyt) and 5-methylcytosine hydrochloride (5-mCyt) were purchased from Sigma-Aldrich (MKBX8310V and MKBQ8997V, USA). The level of genomic DNA methylation was calculated as the percentage of DNA methylation as follows: DNA methylation% = 5 − mCyt/(5 − mCyt + Cyt) × 100%.

### 2.5. Statistical Analysis

All data were expressed as means ± standard error (SEM). Software SPSS 17.0 and OriginPro 8 (OriginLab, Northampton, MA) were used for data analysis. Normality was checked for all data before analysis. Comparisons between different groups were tested using two-sample *t*-test, Kruskal-Wallis ANOVA, Mann-Whitney *U* test, or *χ*^2^ test. The relationships between clinical and biochemical variables, the level of genomic DNA methylation, and TCSS scores were assessed by Spearman correlation analysis. In multiple stepwise regression analysis, TCSS was treated as the dependent variable, and age, duration, UA, CRP, BMI, and eGFR as well as the level of genomic DNA methylation were included as the independent variables. In another multiple stepwise regression analysis, the level of genomic DNA methylation was set as the dependent variable, and age, gender, duration, BMI, FPG, HbA1c, C-peptide, TG, TC, LDL-C, HDL-C, creatinine, UA, BUN, UACR, eGFR, CRP, and TCSS scores were all included as the independent variables. A *p* value less than 0.05 was considered to be statistically significant.

## 3. Results

### 3.1. Clinical and Biochemical Characteristics of the Study Population

**Figure 1:**
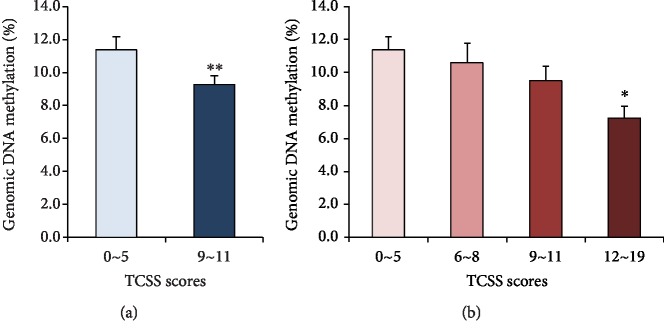
The level of genomic DNA methylation in different groups. (a) The level of genomic DNA methylation was lower in DPN (6~19 points) group compared with non-DPN (0~5 points) group (DPN, *n* = 100; non-DPN, *n* = 86, ^∗^*p* < 0.05, compared with non-DPN, Mann-Whitney *U* test). (b) Dividing the population into 4 subgroups according to TCSS scores, the level of genomic DNA methylation went down as TCSS score increased (TCSS score: 0~5 points, *n* = 86; 6~8 points, *n* = 36; 9~11 points, *n* = 35; 12~19 points, *n* = 29, ^∗^*p* < 0.05, compared with non-DPN, Kruskal-Wallis ANOVA).

A total of 186 patients with type 2 diabetes mellitus were enrolled in the study, including 100 (53.8%) patients in the DPN group and 86 (46.2%) patients in the non-DPN group, divided by the TCSS scores. Clinical and biochemical characteristics are shown in Table 1. Age, duration of DM, triglyceride (TG), total cholesterol (TC), low-density lipoprotein (LDL-C), creatinine, uric acid (UA), blood urea nitrogen (BUN), and C-reactive protein (CRP) were significantly higher in the DPN group (^∗∗^*p* < 0.01, ^∗^*p* < 0.05, compared with non-DPN, Mann-Whitney *U* test). Estimated glomerular filtration rate (eGFR) was much lower in the DPN group (^∗∗^*p* < 0.01, compared with non-DPN, two-sample *t*-test). There were no significant differences in BMI, fasting plasma glucose (FBG), glycated hemoglobin (HbA1c), C-peptide, high-density lipoprotein (HDL-C), and urine microalbumin creatinine ratio (UACR) between two groups (*p* > 0.05). The level of genomic DNA methylation was lower in the DPN group compared with the non-DPN group. The values were 9.244% ± 0.557% and 11.364% ± 0.783%for diabetic patients with DPN (*n* = 100) and without DPN (*n* = 86), respectively (Figure 1(a)) (mean difference -2.12%, ^∗^*p* < 0.05, compared with non-DPN, Mann-Whitney *U* test). We divided the population into 4 subgroups according to TCSS scores, representing non-DPN (<6 points), mild (6~8 points), moderate (9~11 points), and severe DPN (12~19 points), and found that the level of genomic DNA methylation went down as TCSS score increased. The values were 11.364% ± 0.783% for the non-DPN group (*n* = 86), 10.624% ± 1.106% for the mild DPN group (*n* = 36), 9.501% ± 0.873% for the moderate DPN group (*n* = 35), and 7.221% ± 0.726% for the severe DPN group (*n* = 29). However, although the level of genomic DNA methylation decreased with increasing TCSS scores, only the differences between the severe DPN group and non-DPN group were statistically significant (Figure 1(b), ^∗^*p* < 0.05, compared with non-DPN, Kruskal-Wallis ANOVA).

### 3.2. Relationships between Genomic DNA Methylation and TCSS Scores

In order to study the influencing factors of DPN, we applied two correlation analyses. The results of the Spearman correlation analysis among TCSS scores, clinical characteristics, and the level of genomic DNA methylation are shown in Table 2. TCSS was positively correlated with age, duration, UA, UACR, and CRP, and the correlation coefficients (*r* values) were 0.424, 0.556, 0.149, 0.161, and 0.226, respectively (^∗^*p* < 0.05, ^∗∗^*p* < 0.01). In contrast, TCSS was negatively correlated with BMI, eGFR, and the level of genomic DNA methylation, and *r* values were -0.155, -0.353, and -0.278, respectively (^∗^*p* < 0.05, ^∗∗^*p* < 0.01). In the multiple stepwise regression analysis, TCSS was set as a dependent variable and the other 8 variables taken as independent variables. The results showed that the duration of diabetes, the level of genomic DNA methylation, and eGFR were the risk factors of TCSS (Table 3). In the model, *R* = 0.622, adjusted *R*^2^ = 0.372, and *F*(3,119) = 25.048, ^∗∗^*p* < 0.01. Unstandardized coefficients for duration was 0.242 (0.162, 0.322), for genomic DNA methylation was -16.434 (-24.253, -8.615), and for eGFR was -0.027 (-0.043, -0.011), respectively (^∗∗^*p* < 0.01). From the correlation coefficients, genomic DNA methylation was the second risk factor of TCSS after duration of diabetes.

### 3.3. Relationships between Genomic DNA Methylation and Renal Injury

**Figure 2:**
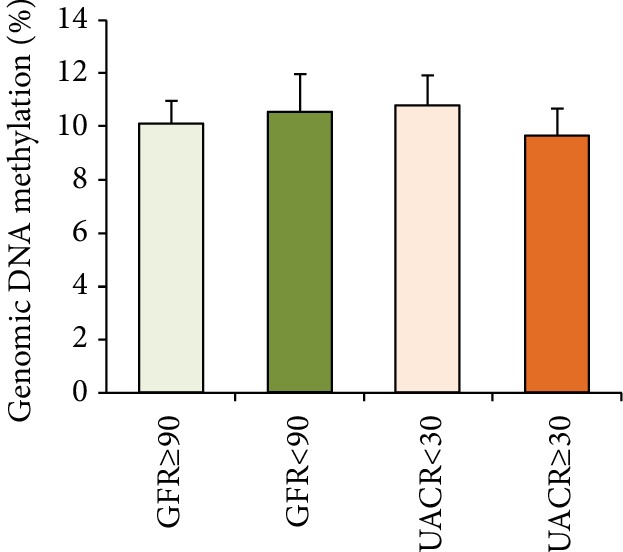
The level of genomic DNA methylation in the nonrenal injury group and renal injury group. The level of genomic DNA methylation did not alter between the two groups. (*p* > 0.05, compared with the renal injury group, Mann-Whitney *U* test).

Then, we wanted to know whether genomic DNA methylation is a specific risk factor for DPN and what factors affect genomic DNA methylation. It is well known that renal injury is another common chronic complication of diabetes. In the study, we divided the population into the nonrenal injury group and renal injury group, according to the different levels of eGFR and UACR, and then compared the levels of genomic DNA methylation between the two groups. The results showed that the levels of genomic DNA methylation did not change significantly between the nonrenal injury group and renal injury group. The levels of genomic DNA methylation in the eGFR ≥ 90 ml/min/1.73 m^2^ group (*n* = 130) and eGFR < 90 ml/min/1.73 m^2^ group (*n* = 56) were 10.094% ± 0.885% and 10.528% ± 1.407%, respectively (Figure 2, *p* = 0.676, Mann-Whitney *U* test). The levels of genomic DNA methylation in the UACR < 30 mg/g group (*n* = 92) and UACR ≥ 30 mg/g (*n* = 94) group were 10.796% ± 1.125% and 9.666% ± 0.997%, respectively (Figure 2, *p* = 0.235, Mann-Whitney *U* test). The difference was not statistically significant, and even the *p* value was greater than 0.2, so we did not conduct multivariate correlation analysis any more.

### 3.4. The Influencing Factors of Genomic DNA Methylation

From the results of the Spearman correlation analysis among genomic DNA methylation and other characteristics, we found that the level of genomic DNA methylation was negatively correlated with TCSS and BMI, and *r* values were -0.278 and -0.176, respectively (data not shown, ^∗^*p* < 0.05, ^∗∗^*p* < 0.01). We next confirmed the influencing factors of genomic DNA methylation with the multiple stepwise regression analysis. In the analysis, the level of genomic DNA methylation was set as a dependent variable and all the clinical variables as independent variables. The results showed that TCSS and BMI were the risk factors of genomic DNA methylation (Table 4). In the model, *R* = 0.392, adjusted *R*^2^ = 0.154, and *F*(2,120) = 10.922, ^∗∗^*p* < 0.01. Unstandardized coefficient for TCSS was -0.5% (-0.9%, -0.2%), and for BMI was -0.6% (-0.9%, -0.2%), respectively (^∗∗^*p* < 0.01).

### 3.5. The Levels of Genomic DNA Methylation in Different Subgroups

All of the above results showed a significant correlation between genomic DNA methylation and TCSS, and the duration of diabetes and BMI was possible influencing factors between them. We sought to further analyze the differences of genomic DNA methylation between the DPN group and non-DPN group in different subgroups according to different levels of duration and BMI. We found that the level of genomic DNA methylation was lower in patients with DPN and diabetic duration more than 5 years. In the subgroup with duration <5 years, the levels of genomic DNA methylation were 10.125% ± 1.581% in patients without DPN and 11.907% ± 2.977% in patients with DPN (Table 5, non-DPN, *n* = 41; DPN, *n* = 16, *p* > 0.05, compared with non-DPN, Mann-Whitney *U* test). While in the subgroup with duration ≥5 years, the levels of genomic DNA methylation were 12.494% ± 1.863% in patients without DPN and 8.737% ± 0.953%in patients with DPN, and the difference between the two groups reached statistical significance (Table 5, non-DPN, *n* = 45; DPN, *n* = 84, ^∗∗^*p* < 0.01, compared with non-DPN, Mann-Whitney *U* test). From Table 5, we also found that regardless of the patient's BMI, the level of genomic DNA methylation was lower in the DPN group. However, only in the subgroup with BMI ≥ 25 kg/m^2^, the difference of genomic DNA methylation between the DPN and non-DPN groups reached statistical significance. In the subgroup of BMI < 25 kg/m^2^, the levels of genomic DNA methylation were 12.855% ± 2.113% in patients without DPN and 10.296% ± 1.414% in patients with DPN (Table 5, non-DPN; DPN, *n* = 53, *n* = 37, *p* > 0.05, compared with non-DPN, Man-Whitney *U* test). While in the subgroup with BMI ≥ 25 kg/m^2^, the levels of genomic DNA methylation were 10.240% ± 1.463% in patients without DPN and 8.058% ± 1.175% in patients with DPN (Table 5, non-DPN, *n* = 49; DPN, *n* = 47, ^∗^*p* < 0.05, compared with non-DPN, Mann-Whitney *U* test).

## 4. Discussion

Diabetic peripheral neuropathy is one of the most common chronic complications of diabetes. In the early stage, there may be no overt symptoms, and as the condition worsens, symptoms such as limb numbness, formication, and tingling and burning pain appear alone or together. In many cases, DPN ultimately leads to serious consequences such as gangrene and amputation with the further development of ischemia and hypoxia in the limbs, seriously affecting the life quality of patients [12, 13]. It is important to study the clinical features and risk factors of DPN for providing more clues for basic research and clinical treatment.

Most studies found that the prevalence of DPN increased with age and duration [12, 14]. In general, more than half of diabetic patients live with different degrees of peripheral neuropathy when the duration is more than 10 years [15]. Long-time hyperglycemia and aging increase production of inflammatory factors, such as IL-6, TNF-*α*, and CRP, which promote the onset of DPN [16]. The results of the present study showed that patients with DPN were older and had longer duration of diabetes when compared with non-DPN patients, and the differences between the two groups were statistically significant. The correlation analysis indicated age and duration of diabetes were the two most important influencing factors of DPN. Our results also showed that CRP in the DPN group was significantly higher than that in the non-DPN group. Spearman correlation analysis further confirmed that CRP was another influencing factor of DPN, which may illustrate an important role of inflammation in the pathogenesis of DPN. In the contrast, the levels of FPG and HbA1C did not show statistical differences between two groups. The reason might be that patients were hospitalized and their blood glucose levels were similar high at admission.

Diabetes is often accompanied by dyslipidemia which increases blood viscosity, affects blood perfusion, forms microthrombus, and eventually reduces blood supply to nerve cells. Lipid metabolism affects energy metabolism and signal transmission in the nervous system [17]. It was reported that oxidized low-density lipoprotein (oxLDL) levels in patients with DPN were higher than in those without neuropathy, and oxLDL could accelerate nerve injury [18] and that the elevated TC, TG, and LDL-C were independent risk factors of DPN [19]. In the present study, TC, TG, and LDL-C levels in the DPN group were significantly higher than those in the non-DPN group. However, Spearman correlation analysis did not further verify the association between dyslipidemia and DPN, which may due to the interference of lipid-lowering drugs that some patients were taking at admission.

To date, the pathogenesis of DPN has not been fully elucidated. It is thought to be caused by multiple comprehensive factors, such as the ischemia and hypoxia in nerves, oxidative stress, hyperactivity of polyol metabolic pathway, activation of protein kinase C, deficiency of growth factors, genetic factors, and immune abnormalities [20]. However, much less is known about epigenetic changes in DPN. Epigenetic regulation, such as DNA methylation, is reported to be an important pathogenesis of diabetic complications. As the disease progresses, hyperglycemia alters the DNA methylation status, which in turn regulates gene expression and promotes the expression of many key molecules, which ultimately leads to diabetic chronic complications [9, 21]. The assessment of DNA methylation status includes analysis of methylation status of specific genes and the whole genomic DNA. Our previous study found that DNA methylation of P2X3 receptor, one of the ligand-gated ion channel, was reduced, promoting the expression of the P2X3 receptor in the dorsal root ganglia and inducing diabetic painful neuropathy in rats [11]. We also found DNA demethylation of cystathionine-*β*-synthetase (CBS), which is the endogenous H_2_S-producing enzyme, induced gastric hypersensitivity in rats with diabetes [22]. Recently, Guo et al. reported the genome-wide DNA methylation profiles of human sural nerve biopsies from subjects with DPN and suggested that epigenetic regulation had an important role in the progression of this prevalent diabetic complication [23]. In the present study, we would like to know whether the level of genomic DNA methylation in white blood cells is related to DPN and become a biomarker for DPN. The results showed that the level of genomic DNA methylation is much lower in patients with DPN, and the higher score of TCSS, the lower degree of whole genomic DNA methylation. Taking TCSS as a dependent variable, the correlation analysis and the multiple stepwise regression analysis both showed genomic DNA methylation level was negatively related to TCSS, and the correlation was statistically significant.

As the results showed in the multiple stepwise regression analysis, eGFR, a good indicator for evaluation of renal injury, was another important influencing factor of TCSS in addition to duration and genomic DNA methylation. Diabetic renal injury and neuropathy have many of the same pathological mechanisms, such as small vessel disease and inflammation [24]. So it was commonly found in clinical practice that the incidence of DPN in patients with diabetic renal injury is high [25] and the low level eGFR is closely associated with DPN [19]. Furthermore, it was reported that aberrant DNA methylation in the proximal tubules was related to diabetic nephropathy [26]. Then, we need to know if genomic DNA methylation is a specific risk factor for DPN, or just a contributing factor to all the chronic complications of diabetes. We divided the population into the nonrenal injury group and renal injury group and compared the levels of genomic DNA methylation between the two groups. The results showed that the levels of genomic DNA methylation did not alter significantly in the renal injury group, when compared to the nonrenal injury group. From the results, we consider that genomic DNA methylation is a relative specific risk factor of DPN. Regrettably, retinopathy, the classic diabetic microangiopathy, was not accounted in the present study due to conditional restrictions.

In order to understand the role of DNA methylation in DPN more clearly, we also analyzed the influencing factors of DNA methylation itself. The results of the correlation analysis and the multiple stepwise regression analysis found that BMI was another negative influencing factor of genomic of DNA methylation preceded only by TCSS. This is consistent with the existing reports. In one of the reports, the researchers studied genome-wide leukocyte DNA methylation variation in 30 clinically healthy young adult monozygotic twin pairs discordant for body mass index. The results showed that significant DNA methylation differences were observed if the heavier cotwins had excessive liver fat, and 91% of the differentially methylated CpGs were less methylated in the DNA from the heavy compared to the lean cotwins. This genome-wide leukocyte DNA demethylation was coupled with insulin resistance and low-grade inflammation [27].

Integrating the influencing factors of TCSS and DNA methylation from all the above analysis, we further studied the differences of genomic DNA methylation between the DPN group and non-DPN group in different subgroups according to different levels of duration and BMI and found that the level of genomic DNA methylation was lower in patients with DPN in the subgroups of diabetic duration ≥5 years and BMI ≥ 25 kg/m^2^. The level of genomic DNA methylation in patients with DPN in the subgroups of BMI < 25 kg/m^2^ had significant downward trend, but the difference did not reach the significance. The results suggested that even with many interfering factors, low level of genomic DNA methylation remains a specific risk factor for patients with DPN.

Combined with our previous researches, we believe that lower DNA methylation status plays an important role in DPN and is a relative specific risk factor for patients with DPN. In addition to the abnormal expressions of the methyltransferases DNMT3a and DNMT3b mentioned in our previous study [22, 28], the specific mechanism may be related to vitamin and folic acid deficiency caused by diabetes. Studies have shown that lower level of folic acid in the blood circulation of diabetic patients is associated with lower genomic DNA methylation [29]. Studies have also shown that long-term supplementation with vitamin B_12_ and folic acid can alter the level of genomic DNA methylation in patients [30]. It is well known that the lack of vitamin B_12_ and folic acid is an important factor in accelerating neuropathy and vitamin B_12_ and folic acid are one-carbon metabolism biomarkers. We speculated that the lack of one-carbon unit in diabetic patients leads to the genomic DNA demethylation and further promotes the expression of key genes, activating more signal pathways leading to DPN.

The present study had some limitations. Firstly, the sample size of the study was small, and the selected patients were regional, so bias factors were present. Secondly, the study was a cross-sectional one, so the relationship between the genomic DNA methylation and the evolution of diabetic neuropathy could not be observed longitudinally. Multicenter, large sample studies are needed. Thirdly, folic acid and vitamins had effects on the level of genomic DNA methylation, but the levels of folic acid and vitamins were not detected in the present study. Finally, some patients with hypertension, hyperlipidemia, and atherosclerosis were enrolled in the study and they were taking appropriate drugs, such as insulin, oral hypoglycemic agents, and antihypertensive drugs, which might affect the results of the study.

## 5. Conclusion

As the above results showed, we found for the first time that low level of genomic DNA methylation is a relative specific risk factor of diabetic peripheral neuropathy in patients with type 2 diabetes.

## Figures and Tables

**Table 1:** Clinical and biochemical characteristics of the study population.

Variables	TCSS < 6 (*n* = 86)	TCSS ≥ 6 (*n* = 100)	*p* value
Gender (M : F)	51 : 35	67 : 33	0.279
Age (y)	56.221 ± 1.298	63.270 ± 1.231	0.001^∗∗^
Duration (y)	5.836 ± 0.551	12.472 ± 0.752	0.000^∗∗^
BMI (kg/m^2^)	25.429 ± 3.615	25.013 ± 2.854	0.391
FPG (mmol/l)	8.637 ± 0.525	9.147 ± 0.496	0.362
HbA1c (%)	8.743 ± 0.289	8.778 ± 0.240	0.597
C-peptide (ng/ml)	1.866 ± 0.109	1.946 ± 0.136	0.839
TG (mmol/l)	1.563 ± 0.168	1.972 ± 0.140	0.003^∗∗^
TC (mmol/l)	4.437 ± 0.135	4.885 ± 0.115	0.005^∗∗^
LDL-C (mmol/l)	2.550 ± 0.096	2.969 ± 0.100	0.003^∗∗^
HDL-C (mmol/l)	1.103 ± 0.031	1.074 ± 0.032	0.277
Creatinine (*μ*mol/l)	63.133 ± 2.223	79.520 ± 3.428	0.004^∗∗^
UA (umol/l)	303.831 ± 10.767	349.704 ± 10.631	0.003^∗∗^
BUN (mmol/l)	5.575 ± 0.212	6.488 ± 0.250	0.012^∗^
eGFR (ml/min/1.73 m^2^)	115.214 ± 3.325	93.660 ± 3.414	0.000^∗∗^
UACR (mg/g)	219.201 ± 121.144	340.638 ± 123.155	0.136
CRP (mg/l)	5.395 ± 0.059	5.615 ± 0.095	0.008^∗∗^

BMI: body mass index; FPG: fasting plasma glucose; HbA1c: glycated hemoglobin A1c; TG: triglycerides; TC: total cholesterol; LDL-c: low-density lipoprotein cholesterol; HDL-c: high-density lipoprotein cholesterol; UA: uric acid; BUN: blood urea nitrogen; eGFR: estimated glomerular filtration rate; UACR: urinary albumin creatinine ratio; CRP: c-reactive protein. Data are means ± SEM, numbers of patients. ^∗^*p* < 0.05, ^∗∗^*p* < 0.01. *p* values for differences between two groups were obtained by two-sample *t*-test, Mann-Whitney *U* test, or *χ*^2^ test.

**Table 2:** Correlation analysis among TCSS scores and other variables.

Variables		Age	Duration	BMI	UA	GFR	UACR	CRP	Genomic DNA methylation
TCSS	*r*	0.424	0.556	-0.155	0.149	-0.353	0.161	0.226	-0.278
	*p*	0.000^∗∗^	0.000^∗∗^	0.035^∗^	0.045^∗^	0.000^∗∗^	0.042^∗^	0.003^∗∗^	0.000^∗∗^

The *r* value indicated the 8 variables, age, duration, BMI, UA, eGFR, UACR, CRP, and genomic DNA methylation, were likely to be related to TCSS (^∗^*p* < 0.05, ^∗∗^*p* < 0.01).

**Table 3:** TCSS as dependent variable in multiple stepwise regression analysis.

	Unstandardized coefficients		Standardized coefficients	*T*	*p* value	95% confidence interval for *B*	
	*B*	Std. error	Beta			Lower bound	Upper bound
Constant	9.117	1.144	—	7.967	0.000^∗∗^	6.851	11.383
Duration	0.242	0.041	0.441	5.975	0.000^∗∗^	0.162	0.322
Genomic DNA methylation	-16.434	3.949	-0.300	-4.162	0.000^∗∗^	-24.253	-8.615
eGFR	-0.027	0.008	-0.253	-3.413	0.001^∗∗^	-0.043	-0.011

In multiple stepwise regression analysis, TCSS, as dependent variable, and the other 8 variables, age, duration, BMI, CRP, UACR, UA, eGFR, and genomic DNA methylation, as independent variables, were included in the same model. Only three variables, duration, genomic DNA methylation, and eGFR, were the risk factors of TCSS (^∗∗^*p* < 0.01).

**Table 4:** Genomic DNA methylation as dependent variable in multiple stepwise regression analysis.

	Nonstandardized coefficients		Standardized coefficients	*T*	*p* value	95% confidence interval for *B*	
	*B*	Std. error	Beta			Lower bound	Upper bound
Constant	0.292	0.048	—	6.118	0.000^∗∗^	0.198	0.387
TCSS	-0.005	0.002	-0.300	-3.564	0.001^∗∗^	-0.009	-0.002
BMI	-0.006	0.002	-0.276	-3.283	0.001^∗∗^	-0.009	-0.002

In multiple stepwise regression analysis, genomic DNA methylation, as dependent variable, and all the clinical variables, as independent variables, were included in the same model. Two variables, TCSS and BMI, were the influencing factors of genomic DNA methylation (^∗∗^*p* < 0.01).

**Table 5:** Genomic DNA methylation between the DPN and non-DPN groups in different subgroups.

Variables		Duration (y)		BMI (kg/m^2^)	
	Subgroups	<5	≥5	<25	≥25
Genomic DNA methylation (%)	TCSS < 6	10.125 ± 1.581	12.494 ± 1.863	12.855 ± 2.113	10.240 ± 1.463
	TCSS ≥ 6	11.907 ± 2.977	8.737 ± 0.953	10.296 ± 1.414	8.058 ± 1.175
*p* value		0.217	0.009^∗∗^	0.089	0.044^∗^

Divided the population into subgroups according to the different levels of duration and BMI and then compared the difference of the level of genomic DNA methylation between the DPN and non-DPN groups in those subgroups. The level of genomic DNA methylation was lower in patients with DPN and diabetic duration more than 5 years (^∗∗^*p* < 0.01, compared with non-DPN, Mann-Whitney *U* test) and was also significantly lower in patients with DPN and BMI ≥ 25 kg/m^2^ (^∗^*p* < 0.05, compared with non-DPN, Mann-Whitney *U* test). The level of genomic DNA methylation in patients with DPN in the subgroups of BMI < 25 kg/m^2^ had significant downward trend, but the difference was not significant (*p* > 0.05, compared with non-DPN, Mann-Whitney *U* test).

## Data Availability

All data generated or analyzed throughout this study are included in this published article and its supplementary information files.

## Abbreviations

DPN: Diabetic peripheral neuropathies
TCSS: Toronto Clinical Scoring System
LC-MS/MS: Liquid chromatography-tandem mass spectrometry
BMI: Body mass index
FPG: Fasting plasma glucose
HbA1c: Glycated hemoglobin
TC: Total cholesterol
TG: Triglyceride
HDL-C: High-density lipoprotein
LDL-C: Low-density lipoprotein
UA: Uric acid
BUN: Blood urea nitrogen
CRP: C-reactive protein
UACR: Urine microalbumin creatinine ratio
eGFR: Estimated glomerular filtration rate.

## Supplementary Materials

Supplementary Materials Supplementary Table 1: the specific conditions for LC-MS/MS analysis. Supplementary Figure 1: the typical chromatographic peaks.
