# Supplementary material for: The Association between Genomic DNA Methylation and Diabetic Peripheral Neuropathy in Patients with Type 2 Diabetes Mellitus
Source: J Diabetes Res. 2019 Nov 3;2019:2494057. doi: 10.1155/2019/2494057 (PMC6875377; doi:10.1155/2019/2494057)
Supplement: Supplementary Materials — Supplementary Table 1: the specific conditions for LC-MS/MS analysis. Supplementary Figure 1: the typical chromatographic peaks. [file 2494057.f1.pdf]

**The specific conditions of HPLC**							
Chromatographic column	XTerra® RP18（4.6×150 mm, 3.5 μm）						
Pre-column	Phenomenex Security Guard ™ C_{18}（4 × 3.0 mm）						
TEM of column	30℃						
Mobile phase	A：Water B：Methanol						
	Time	A%	B%	Flow rate (mL/min)			
	0	97	3	0.5			
	5	97	3	0.5			
	6	0	100	0.5			
	7.5	0	100	0.5			
	8	97	3	0.5			
	15	97	3	0.5			
Running time	15 min						
Sampling volume	10 μL						
TEM of autosampler	4℃						
**The specific conditions of MS**							
Ion source	ESI						
Ionic polarity	Positive						
Scan patterns	MRM						
Ion	Precursor Ion（Da）	Product Ion（Da）	Dwell Time（msec）	DP（V）	EP（V）	CE（V）	CXP（V）
dC	228.2	112.1	200	40	10	15	10
5-mdC	242.2	126.1	200	40	10	22	10
cyt	112.1	95.1	200	114	10	20	10
5mcyt	126.1	109.1	200	114	10	20	10
dG	268.2	152.1	200	40	10	22	10
CAD	8 psi						
CUR	30 psi						
GS1	50 psi						
GS2	50 psi						
IS	3500 V						
TEM	550℃						

Supplementary Table 1. The specific conditions for LC-MS/MS analysis.

ESI: electron spray ionization. MRM: multiple reaction monitoring. DP: declustering potential. EP: entrance potential. CE: collision energy. CXP: collision cell exit potential. CAD: collision gas CUR: curtain gas. GS1: gas 1. GS2: gas 2. IS: ion spray voltage. TEM: temperature.

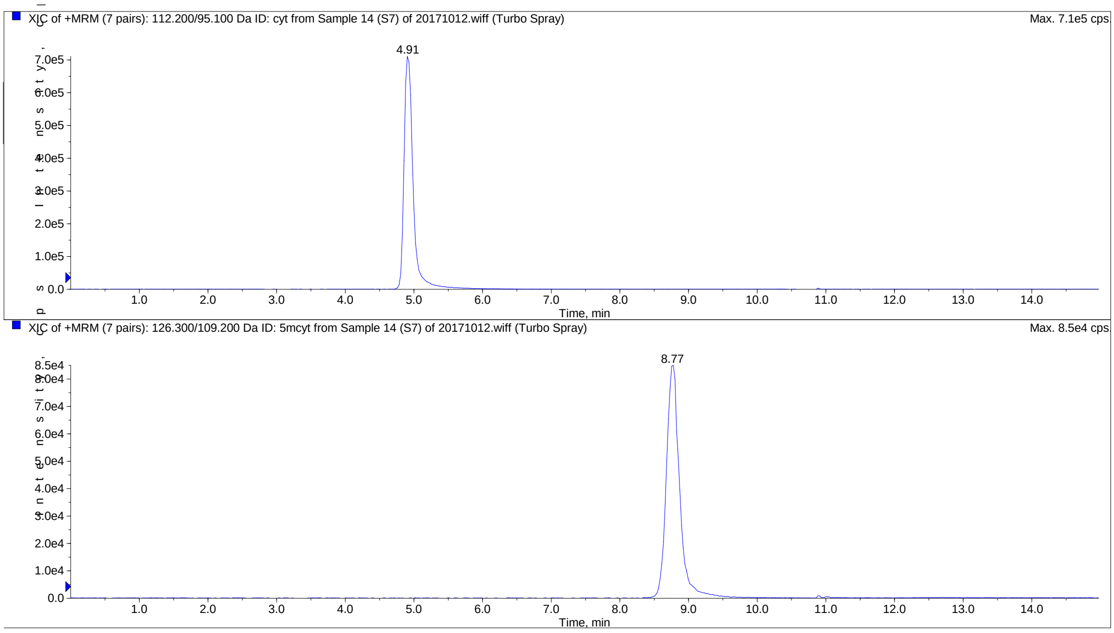

Supplementary Figure 1.The typical chromatographic peaks.

The typical chromatogram of solution spiked with cyt and 5mcyt.
